# Biochemical Validation of a Self-Administered Food Frequency Questionnaire to Assess Diet Using Carotenoids and Vitamins E and D in Male Adolescents in Spain

**DOI:** 10.3390/antiox10050750

**Published:** 2021-05-08

**Authors:** Leyre Notario-Barandiaran, Eva-María Navarrete-Muñoz, Desirée Valera-Gran, Elena Hernández-Álvarez, Encarnación Donoso-Navarro, Sandra González-Palacios, Manuela García-de-la-Hera, Mariana F. Fernández, Carmen Freire, Jesús Vioque

**Affiliations:** 1 Alicante Institute for Health and Biomedical Research, ISABIAL-UMH, 03010 Alicante, Spain; lnotario@umh.es (L.N.-B.); enavarrete@umh.es (E.-M.N.-M.); sandra.gonalezp@umh.es (S.G.-P.); manoli@umh.es (M.G.-d.-l.-H.); 2 Nutritional Epidemiology Unit, University Miguel Hernandez, 03550 Alicante, Spain; 3 InTeO Research Group, Department of Pathology and Surgery, Miguel Hernández University, 03550 Alicante, Spain; dvalera@umh.es; 4 Department of Clinical Biochemistry, Puerta de Hierro University Hospital Majadahonda, 28222 Madrid, Spain; mhalvarez@salud.madrid.org (E.H.-Á.); encarnacion.donoso@salud.madrid.org (E.D.-N.); 5 Spanish Consortium for Research on Epidemiology and Public Health (CIBERESP), Institute of Health Carlos III, 28009 Madrid, Spain; marieta@ugr.es (M.F.F.); cfreire@ugr.es (C.F.); 6 Biosanitary Research Institute of Granada (ibs.GRANADA), 18012 Granada, Spain; 7 Department of Radiology, School of Medicine and Center for Biomedical Research, University of Granada, 18071 Granada, Spain

**Keywords:** carotenoid intake, vitamin E, vitamin D, fruit and vegetable intake, food frequency questionnaire, adolescence, biochemical validity, nutritional biomarker

## Abstract

Reliable tools to evaluate diet are needed, particularly in life periods such as adolescence in which a rapid rate of growth and development occurs. We assessed the biochemical validity of a self-administered food frequency questionnaire (FFQ) in a sample of Spanish male adolescents using carotenoids and vitamin E and D data. We analyzed data from 122 male adolescents aged 15–17 years of the INMA-Granada birth cohort study. Adolescents answered a 104-item FFQ and provided a non-fasting blood sample. Mean daily nutrient intakes and serum concentration were estimated for main carotenoids (lutein-zeaxanthin, β-cryptoxanthin, lycopene, α-carotene and β-carotene), vitamins E and D and also for fruit and vegetable intake. Pearson correlation coefficients (*r*) and the percentage of agreement (same or adjacent quintiles) between serum vitamin concentrations and energy-adjusted intakes were estimated. Statistically significant correlation coefficients were observed for the total carotenoids (*r* = 0.40) and specific carotenoids, with the highest correlation observed for lutein–zeaxanthin (*r* = 0.42) and the lowest for β-carotene (0.23). The correlation coefficient between fruit and vegetable intake and serum carotenoids was 0.29 (higher for vegetable intake, *r* = 0.33 than for fruit intake, *r* = 0.19). Low correlations were observed for vitamin E and D. The average percentage of agreement for carotenoids was 55.8%, and lower for vitamin E and D (50% and 41%, respectively). The FFQ may be an acceptable tool for dietary assessment among male adolescents in Spain.

## 1. Introduction

Interest in developing tools to evaluate diet has increased in recent years, since it has been shown how diet can have a very important influence on the development of certain chronic diseases such as obesity, type 2 diabetes and cardiovascular diseases [1,2,3,4]. Adolescence is a period of vital importance for development, and diet may play an important role in nutritional status and health [5]. The consumption of fruit and vegetables has shown beneficial health effects probably due to the antioxidant and anti-inflammatory properties of some vitamins such as carotenoids and vitamin E, which may help to protect cellular systems from oxidative damage and also lower the risk of chronic diseases [6]. Other nutrients such as vitamin D play a fundamental role in the metabolism and absorption of minerals in adolescence, the health of the bones and the preservation of bone calcium homeostasis [7]. A poor vitamin D status has also been related to a higher risk of mortality and of some cardiovascular diseases and infectious diseases [8] [9]. Therefore, precise and accurate methods are needed for dietary assessment in this period. 

The Food Frequency Questionnaire (FFQ) has been shown to be very useful in epidemiological studies to capture data on the usual diet to estimate nutrient intakes and food patterns for a longer period of time and to establish diet–disease associations [10,11]. In addition, the FFQ is a time-effective method due to its easy administration, low cost and easy processing [11]. However, FFQs are often criticized due to their susceptibility to measurement errors that can be in the form of random and/or systematic errors [11,12]. These types of errors can be minimized by increasing the number of days, = repeating measurements when food records or 24 h recalls are used, or by using instruments such as FFQ properly validated or calibrated against other reference methods [13]. In this sense, biochemical biomarkers may act as an objective measure of nutrient intakes, and therefore, they may be a good reference method to validate FFQs, as the error sources of the two methods are independent of each other [14]. Furthermore, some authors have suggested that biomarkers should be used to complement the FFQs rather than substitute them [15]. 

To date, numerous FFQs have been developed in different countries, especially for adult populations [16,17,18]; however, in adolescents, the number of validated FFQs with an acceptable reproducibility and validity is still small, and validation against biochemical biomarkers is scarce [19,20,21]. In the last decade, we have developed and validated FFQs for pregnant women and children of different ages in the context of the INMA (Infancia y Medio Ambiente—Environment and Childhood) Project, a prospective birth cohort study in Spain [22,23,24]. More recently, we evaluated the reproducibility and validity of the FFQ used in a self-administered way in a small sample of adolescents included in this study, using as the reference method the two 24 h dietary recalls (24hDR), and it showed low to moderate correlations adequate to support the use of the self-administered FFQ [25]. Thus, in this study, we assessed the biochemical validity of a previously validated FFQ by comparing the self-reported consumption of fruit and vegetables and the estimated intakes of carotenoids and vitamins E and D against their serum concentration in a wider sample of Spanish male adolescents. 

## 2. Materials and Methods

### 2.1. Study Population

**Figure 1:**
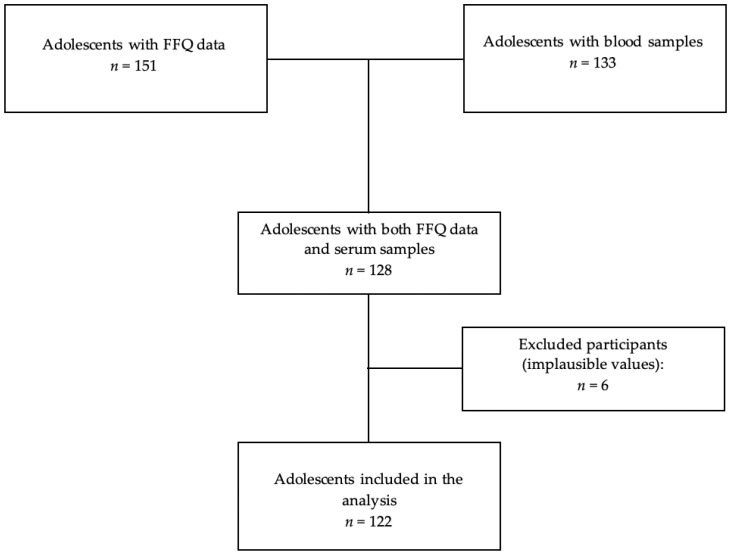
Flowchart of the study population describing the selection process.

This study included 155 male adolescents aged 15 to 17 years belonging to the INMA-Granada cohort who participated in their clinical follow-up visit in 2017–2019 [26,27]. The INMA Project is a multicenter birth prospective cohort study designed to evaluate the effect of environmental exposures and diet during pregnancy, childhood and adolescence in seven Spanish regions with similar protocols [28]. At the follow-up visit, the adolescents received specific instructions to answer the FFQ in a self-administered way and also with the support of fieldworkers. In the same visit, a blood sample was collected from each participant. A total of 151 adolescents completed the FFQ and 133 provided the blood sample (Figure 1). Even though 128 participants had data for both the FFQ and serum samples, the final analysis was based on 122 adolescents since 6 of them were excluded due to implausible values for mean daily energy intake (2 < 800 and 4 > 4000 kcal/day) [11,29]. An informed consent form was signed by the parents of all participants before collecting personal information and biological samples. The study followed the principles of the declaration of Helsinki and was approved by the Ethics Committee of San Cecilio University Hospital of Granada and Miguel Hernández University (Ethical approval number from Granada Hospital CEI 3/2017); parents also provided written informed consent for all participants.

### 2.2. Dietary Assessment: Semi-Quantitative Food Frequency Questionnaire

We used a 104-item semi-quantitative FFQ to assess the usual adolescent’s diet. The FFQ was an adaptation of other FFQs previously validated [22,23] and had a similar structure to the Harvard questionnaire [30]. To adapt the new FFQ for adolescents, we incorporated food items and portion sizes appropriate for adolescents aged 15 to 17 years. The adolescents were provided with instructions to respond to the FFQ in a self-administered way. They reported the average frequency of consumption for each food item in the FFQ during the previous year. The questionnaire had nine possible answers, from “never once or less than once a month” to “six or more times a day”. To estimate nutrient values and total energy intake, we used Spanish published sources and the food composition tables of the US Department of Agriculture (USDA) publications [31,32]. In order to obtain average daily nutrient intakes, we multiplied the frequency of consumption of each food by the nutrient composition of the portion serving size specified in the FFQ. Supplement use was not taken into account to estimate nutrient intake due to the very low percentage of adolescents taking supplements in our study.

### 2.3. Nutritional Biomarkers

Non-fasting blood samples were drawn between 4 and 8 p.m. at the San Cecilio University Hospital (Granada). Samples were centrifuged for five minutes, and afterwards, serum samples were stored at −80 °C. Samples were finally sent to the General Biochemistry Laboratory of the Hospital Universitario Puerta de Hierro Majadahonda for biochemical analysis, using the standardized protocol to keep the samples frozen. Determinations of vitamins and carotenoids were performed according to routine quality-controlled standard methods [22,23,33]. Serum levels of carotenoids (μg/100 mL) and vitamin E (μg/100 mL) and D (nmol/L) were simultaneously measured by ultra-fast liquid chromatography [34]. For lutein and zeaxanthin, the serum values were combined because in the food composition tables, the information for these carotenes is grouped. The short- and long-term precision and accuracy of the analytical methodology were guaranteed since the lab General Biochemistry and Hematology was participating in the Fat-Soluble Quality Assurance Programme conducted by the National Institute of Standards and Technology (NIST; Gaithersburg, MD, USA), in the Vitamin D External Quality Assessment Scheme conducted by the Charing Cross Hospital (DEQAS, London, UK), and serum concentrations of high-density lipoprotein (HDL) and low-density lipoprotein (LDL) (mg/dL) were determined by colorimetric enzymatic methods using a Roche/Hitachi analyzer at the Instituto de Investigación Biosanitaria de Granada (ibs.GRANADA).

### 2.4. Covariates

The date of the interview and health examination was registered. Information on personal characteristics was collected during the interviews. The height and weight of adolescents were measured without shoes and in light clothing using an electronic scale (TANITA model 354, Seca Corporation, Hamburg, Germany). Body mass index (BMI) was calculated as weight in kilograms divided by the square of height in meters, and we categorized BMI according to the specific cutoffs proposed by the International Obesity Task Force (IOTF) [35]. 

### 2.5. Statistical Analysis

We calculated means and standard deviations (SD) for total nutrient intakes and their serum concentrations. All nutrients were natural log-transformed prior to analysis to improve their normality. Energy-adjusted intakes were estimated using the residual method, where each nutrient is regressed on total calories, and the population mean was then added back to the calculated residuals [36]. Since most carotenoids are transported in plasma lipoproteins, serum concentrations of carotenoids and vitamins were also adjusted for total cholesterol concentration using the residual method as well. 

To assess the biochemical validity of the FFQ, we estimated Pearson correlation coefficients between the energy-adjusted dietary intakes from the FFQ and their respective serum concentrations for the main carotenoids (lutein-zeaxanthin, lycopene, β-cryptoxanthin, α-carotene and β-carotene) and for the vitamin E and D. We also calculated the percentage of agreement as to the proportions of individuals who were classified correctly into the same or adjacent quintile. We also repeated the correlation analyses according to the season of the year grouping, spring–summer and autumn–winter, in two categories. Data analyses were performed with STATA^®^, version 16 [37], and *p*-values < 0.05 were considered statistically significant (coefficient correlations higher than 0.20 resulted in statistically significant results in this study).

## 3. Results

The main characteristics of the study participants are shown in Table 1. The mean age of the adolescents and their mothers was 16.2 and 39.7 years, respectively. Regarding the energy and macronutrients intake from the FFQ, the adolescents had a mean daily energy intake of 3044.7 kcal/day, 135.5 g/day for proteins, 347.8 g/day for carbohydrates and 127.3 g/day for total fat.

Pearson correlation coefficients between the nutrient intakes estimated from the FFQ and their serum concentrations are presented in Table 2. For log-transformed intakes, the average of correlation coefficients (*r*) for carotenoids was 0.39, ranging from 0.22 (β-carotene) to 0.35 (lutein-zeaxanthin). Correlations between energy-adjusted intakes and their cholesterol-adjusted serum concentration were similar to the log-transformed values, although the average correlation coefficient for energy-adjusted carotenoid intakes was higher, *r* = 0.40. Correlations ranged from 0.23 (β-carotene) to 0.42 (lutein-zeaxanthin), all of them statistically significant (*p* < 0.05). Correlations between energy-adjusted intakes and serum levels of vitamin E and D were much lower, 0.08 and 0.12 (Table 2). 

Significant correlation coefficients were observed between the energy-adjusted intake of fruit and vegetables and serum concentration of total carotenoids, *r* = 0.29. The correlations were higher for vegetable intake (*r* = 0.33) than for fruit intake (*r* = 0.19). Correlations between fruit and vegetable intakes and serum vitamin E and D levels were higher than those observed for nutrient intakes, *r* = 0.15 and *r* = 0.22, respectively (Table 2). The serum vitamin E correlation coefficient was higher for vegetable intake (*r* = 0.22), whereas correlation coefficient for serum vitamin D was higher for fruit intake (*r* = 0.23), both statistically significant (*p* < 0.05). 

When we repeated the analysis stratifying by season of the year, using two categories, we found much better correlations for carotenoids, vitamin E and fruit and vegetable intakes for the spring–summer seasons than for the autumn–winter seasons, although for vitamin D, the correlations were higher in the cold seasons (Table 3). 

The average percentage of agreement between carotenoid intake and serum concentrations of biochemical biomarkers was 55.8%, ranging from 50.0% for α-carotene to 58.3% for lutein-zeaxanthin, β-cryptoxanthin and lycopene. The agreements were lower for vitamin E and D, 50.0% and 41.7%, respectively.

## 4. Discussion

This study shows significant correlations between carotenoids serum concentrations and the intake of fruits and vegetables and carotenoids estimated using an FFQ. Overall, these results would support the relative validity of the FFQ and the adequacy of this dietary assessment method to assess diet among adolescents in Spain, despite the low correlations observed for vitamins E and D. To our knowledge, this is the first biochemical validation study of an FFQ that is suitable to be self-administered among male adolescents and reinforces the validity and reproducibility previously reported for the same FFQ after comparing FFQ intakes with those of two 24 h dietary recalls [25]. 

The correlation coefficients observed in our study are in general in the range of those observed in the literature for the same vitamin intakes and their serum concentrations [38]. The correlations found in our study between carotenoids intakes and their serum concentration were slightly higher than those observed in a study carried out in Norway with 261 participants aged 8–14 years, where it obtained an average correlation for carotenoids of 0.32 [39]. In another study carried out in Connecticut, USA [40], the average correlation for carotenoids was similar (*r* = 0.35) to the average observed in our study, although this study was carried out in an older population. On the other hand, our correlation coefficients were better than those reported in two studies performed in adolescents. One study from Brazil [41], with a correlation coefficient of 0.21, was observed between the intake of carotenes from the FFQ and the serum β-carotene values, and another study, with 285 adolescents aged 12–17 years from three US cities, with an average correlation for carotenoids of 0.23, ranging from 0.08 for lycopene to 0.38 for β-cryptoxanthin [42]. In a previous study that we carried out with elderly subjects from a cross-sectional European study in Spain, slightly lower correlation coefficients were also observed for carotenoids [33]. 

We also estimated positive correlations between carotenoids serum concentration and intake of fruit and vegetables. The correlation was higher for vegetable intake (0.33) than for fruit intake (0.19). Higher correlations have also been observed for vegetables in other studies [43,44], although better correlations have been reported for fruit intake by others [45]. It is unclear the reasons for these differences, although it has been pointed out that vegetables may be a better source for some carotenoids such as lutein, whereas fruits may be a better source for other carotenoids (e.g., B-cryptoxanthin) [46]. In fact, the coefficient correlation between vegetable intake and the lutein–zeaxanthin serum concentration was the highest in our study, i.e., *r* = 0.47 (data not shown). Overall, these results would support that serum carotenoids are a good biomarker of fruit and vegetable consumption. In fact, when we split the correlation analyses by season of the year, we observed much better correlations between serum level and intakes of carotenoids and fruit and vegetables in the spring–summer seasons, which may reinforce the biochemical validity of the FFQ. 

The correlation coefficients between vitamin E and D intakes and their serum concentration in our study were much lower than those for carotenoids. These lower correlations have also been reported in the literature. In a study carried out with 68 children, a null correlation was reported for vitamin D [47]. Low correlations have also been reported in studies with adult populations [18,29,34,35]. These poor correlations for vitamin D may be related to the fact that this vitamin is mainly synthesized in the skin by sun exposure, particularly when vitamin D intake is low, thus being a better indicator of dietary intake in individuals with low sun exposure [48]. The better correlation found between serum and nutrient vitamin D in our study in the cold seasons may in part support that serum level of this vitamin may also be an acceptable marker when sun exposure is low. For vitamin E, it has been suggested that serum concentration may not be a good marker for the usual nutrient intake among adolescents and that the adipose tissue may better represent usual vitamin E intake [48]. In addition, several studies have shown lower serum levels of vitamin E in obese subjects compared to normal-weight subjects due to the sequestration of α-tocopherol in adipose tissue [49,50]. When we stratified by BMI and repeated the correlation analyses, we observed higher correlations of vitamin E in adolescents with normal weight (*r* = 0.17) than those with overweight or obesity, *r* = 0.02 (data not shown). The correlations for vitamin E were also better in the spring–summer than in the autumn–winter seasons, particularly for vegetable intake and serum vitamin E concentration, which may relate to the higher availability of food sources of vitamin E during these seasons and, consequently, better reporting by adolescents when completing the FFQ. It is also of note that the correlations between serum concentration of vitamin E and D and fruit and vegetable intake were higher than the observed for nutrient intakes of these vitamins, which may denote that the serum concentration of vitamin E and D is a better marker of fruit and vegetable intake than the specific intakes estimated for these vitamins by the FFQ. 

This study may have some limitations. The sample size could be one of the limitations; however, the most common sample size recommended is 100–110 participants [11], and it has been suggested in other studies that a sample size of 50 participants is acceptable [16], although sample sizes close to 110–120 participants are recommended, as in our case [11]. Dietary data were mostly collected using an FFQ in a self-administered way, and this may cause bias as it has been reported that adolescents may misreport their energy intake [51]. However, we did not find evidence of any differential reporting with respect to specific foods or total energy intake. Another limitation may relate to the fact that the FFQ was validated only among male adolescents, and caution should be taken if the questionnaire is to be used with female adolescents. The high variability in crude nutrient intakes (high SD) observed in our study for carotenoids is usually found in nutritional population studies mainly related to the high intra- and interindividual variation. We used natural log-transformed and energy-adjusted nutrient estimates to improve their normality and the performance of statistical tests. There are also several strengths in this study, but the main one is that we have used a reference method to validate the FFQ (biochemical markers) that may be considered more objective than other reference methods such as 24hDR or dietary records and exempt from certain types of errors they may encounter. Additional strengths are the use of standard protocols to collect and analyze blood samples and specific instructions for completing the FFQ. In addition, the reproducibility and validity of the FFQ used in this study were previously evaluated against two non-consecutive 24hDR [25]. The study participants are a subsample of a population-based birth cohort study, the INMA project, with great relevance and representativeness in Spain, and that would make the results obtained more generalizable in the male adolescent population. 

## 5. Conclusions

In conclusion, this study supports that our FFQ is suitable to be self-administered and may be an acceptable dietary assessment method to be used in epidemiologic studies among male adolescents aged 15–17 years in Spain, particularly for antioxidant nutrients such as carotenoids and their main food sources (fruit and vegetables). The poor correlations found between intake of vitamin E and D and their serum concentrations, also reported in other validation studies, should be further investigated. Thus, despite the low correlation coefficients observed for vitamin E and D, the FFQ can be recommended for dietary assessment among other adolescent populations in Spain. It is freely accessible and downloadable at http://epinut.edu.umh.es/bibliodieta/ (accessed on 19 April 2021).

## Figures and Tables

**Table 1:** Characteristics of INMA-Granada cohort study participants (*n* = 122).

Personal Characteristics	Descriptive Statistic
Age (in years), mean (SD)	16.2 (0.42)
Body mass index (kg/m^2^), IOTF ^1^ categories, *n* (%)	
Normoweight, 16.0–22.3 kg/m^2^	64 (52.5)
Overweight, 22.4–44.0 kg/m^2^	58 (47.5)
Energy intake (kcals/day), mean (SD)	3044.7 (1675)
Protein intake (g/day), mean (SD)	135.5 (75)
Carbohydrate intake (g/day), mean (SD)	347.8 (205)
Total fat intake (g/day), mean (SD)	127.3 (73)

^1^ IOTF, International Obesity Task Force (Cole, 2000).

**Table 2:** Mean daily intakes and serum concentration of vitamins and correlation coefficients between intakes from the FFQ and serum vitamins.

Nutrient Intakes (Units/Day)	FFQ Intakes	Serum Concentration (μg/100 mL)	Pearson Correlation Coefficient *		% Agreement
	Mean (SD)	Mean (SD)	r	r adj.	
**Carotenoids**					
**Lutein–Zeaxanthin (μg)**	2967.4 (2891)	12.1 (3.8)	0.35	0.42	58.3
**β-Cryptoxanthin (μg)**	347.2 (292)	13.2 (8.7)	0.33	0.26	58.3
**Lycopene (μg)**	4502.9 (3510)	33.8 (15.2)	0.31	0.34	58.3
**α-Carotene (μg)**	1001.4 (1135)	7.0 (2.7)	0.33	0.28	50.0
**β-Carotene (μg)**	4215.5 (3758)	20.6 (11.3)	0.22	0.23	54.2
*Average correlations for carotenoids*			0.39	0.40	55.8
*Fruit and vegetable intake* vs. *serum carotenoids*	594.0 (579)	86.8 (28.3)	0.28	0.29	50.0
*Vegetable intake* vs. *serum carotenoids*	221.0 (214)	86.8 (28.3)	0.24	0.33	50.0
*Fruit intake* vs. *serum carotenoids*	373.0 (440)	86.8 (28.3)	0.21	0.19	50.0
**Vitamin E (mg)**	10.4 (6.8)	933 (156)	−0.06	0.08	50.0
*Fruit and vegetable intake* vs. *serum vitamin E*	594.0 (579)	933 (156)	0.15	0.15	54.2
*Vegetable intake* vs. *serum vitamin E*	221.0 (214)	933 (156)	0.10	0.22	66.7
*Fruit intake* vs. *serum vitamin E*	373.0 (440)	933 (156)	0.08	0.07	45.8
**Vitamin D (μg)**	8.3 (6.0)	70.3 (24)	0.05	0.12	41.7
*Fruit and vegetable intake* vs. *serum vitamin D*	594.0 (579)	70.3 (24)	0.19	0.22	50.0
*Vegetable intake* vs. *serum vitamin D*	221.0 (214)	70.3 (24)	0.12	0.13	50.0
*Fruit intake* vs. *serum vitamin D*	373.0 (440)	70.3 (24)	0.22	0.23	45.8

r: coefficient correlations for log-transformed nutrient intakes and serum carotenoids and vitamin levels. r adj: coefficient correlations for energy-adjusted nutrient intakes and cholesterol-adjusted serum carotenoids and vitamins. * Coefficients higher than 0.20 resulted in statistically significant results in this study (*p* < 0.05).

**Table 3:** Correlation coefficients between energy-adjusted intakes from FFQ and serum concentration of vitamins by season of the year in adolescents aged 15–17 years of the INMA-Granada study (*n* = 122).

	Seasons of the Year	
	Spring–Summer *n* = 45	Autumn–Winter *n* = 77
**Carotenoids**		
**Lutein–Zeaxanthin (μg)**	0.46	0.43
**β-Cryptoxanthin (μg)**	0.25	0.28
**Lycopene (μg)**	0.59	0.15
**α-Carotene (μg)**	0.33	0.23
**β-Carotene (μg)**	0.36	0.16
*Average correlations for all carotenoids*	0.57	0.32
*Fruit and vegetable intake* vs. *serum carotenoids*	0.33	0.27
*Vegetable intake* vs. *serum carotenoids*	0.50	0.22
*Fruit intake* vs. *serum carotenoids*	0.14	0.26
**Vitamin E (mg)**	0.16	0.06
*Fruit and vegetable intake* vs. *serum vitamin E*	0.15	0.13
*Vegetable intake* vs. *serum vitamin E*	0.35	0.14
*Fruit intake* vs. *serum vitamin E*	−0.01	0.08
**Vitamin D (μg)**	−0.02	0.22
*Fruit and vegetable intake* vs. *serum vitamin D*	0.24	0.19
*Vegetable intake* vs. *serum vitamin D*	0.30	0.02
*Fruit intake* vs. *serum vitamin D*	0.14	0.28

Coefficients higher than 0.20 were statistically significant (*p* < 0.05).

## Data Availability

The data presented in this study are available on request from the corresponding author.
